# Editorial: Remarkable Structural Anomalies in Congenital Heart Disease: A Collection of Case Reports in Pediatric Cardiology

**DOI:** 10.3389/fcvm.2022.871222

**Published:** 2022-03-24

**Authors:** Matthias Sigler

**Affiliations:** Pediatric Cardiology and Intensive Care, Georg-August-University Göttingen, Göttingen, Germany

**Keywords:** cardiovascular morphology, congenital heart defect (CHD), case report, cardiac imaging anatomy, aneurysm (abdominal aorta thoracic aorta)

Congenital cardiovascular malformations are the commonest birth defects at all. They have a high prevalence in almost 1% of all newborns (1). The spectrum of the defects is huge. Despite all efforts and advances in imaging techniques and other diagnostic tools, some forms of rare and complex defects remain a challenge and it is difficult to obtain the correct diagnosis and treat them effectively. In this collection of case reports, a variety of remarkable anomalies is displayed, representing different groups of rare malformations affecting the cardiovascular system.

Thomas et al. report on a patient with known genetic risk for vascular aneurysms (Fibulin-4 deficiency) who developed extreme dilatations of all parts of the thoracic aorta. Despite the high risk for a fatal outcome, their group were able to successfully reconstruct and partially replace the aorta surgically in a 7-year-old patient.

Another patient with a giant aneurysm of the thoracic aorta and also effective surgical intervention is displayed by Li et al. In contrast to the former case, the dilatation was due to pseudo-aneurysm formation secondary to aortic cannulation during former surgery for a ventricular septal defect in combination with suspected bacterial aortitis. The case of a rare variant of sinus of Valsalva aneurysm is reported by Gong et al. who were able to control a significant left-to-right shunt between a ruptured aneurysm of the right coronary sinus and the right ventricle. Shunt closure was achieved by interventional implantation of a ventricular septal defect occluder.

Coronary artery fistulae are the commonest congenital anomaly of the coronary artery system. They almost always drain to right heart structures and become hemodynamically relevant in only a few patients. Surgical intervention was indicated in the case of a huge isolated fistula between the right coronary artery and right ventricle presented by Yuan et al. in a newborn that became symptomatic as early as in the third week of life.

The urgent need for a careful and correct diagnosis prior to therapeutic interventions is demonstrated by the case of a 9 month old patient with a pulmonary artery sling. It was only after surgical re-implantation of the left pulmonary artery that significant tracheal stenosis (which usually accompanies pulmonary artery sling) was identified as the cause for failure to wean from ventilator support (Shi et al.). A less uncommon anomaly is the separate drainage of hepatic veins directly into the right atrium. In a patient presented by Luo and Bu this anomaly was missed prior to total cavopulmonary connection with an extracardiac conduit. The situation came to attention only when the patient was found cyanotic in the postoperative course. This patient had to undergo redo surgery due to inadequate diagnostic workup preoperatively.

An example of a rare venous malformation, namely a retro aortic route of the innominate vein in combination with total anomalous pulmonary venous return, is displayed by Ifuku et al. who speculate on an explanation for the abnormal route based on developmental considerations.

Even neoplasms can affect the heart of the newborn. Vakrilova et al. report on an extremely rare case of a congenital malignant cardiac sarcoma in a newborn that at first presented with the clinical picture of neonatal infection. It was because of progressive deterioration despite anti-infective treatment that echocardiographic workup revealed the tumor, which was surgically excised subsequently.

In summary, these cases report either extremely rare malformations of the heart and the vascular system or unusual but sometimes only unforeseen clinical courses of congenital cardiovascular anomalies. Different imaging techniques have been employed including computed tomography, echocardiography, or angiography. These and other imaging techniques become better and better with improvements made both in data acquisition and data processing. But still, we always have to interpret what we see in the images we produce.

For understanding cardiovascular malformations and to correctly interpret imaging data, the basis is still a profound knowledge in cardiovascular morphology and gaining as much experience as possible with rare abnormalities of the heart and vessels. The latter makes it so important to read and learn from case reports—as you are about to do by going through this collection of remarkable cases. Readers will only recognize what you have seen before or what you have at least heard or read about. On the other hand, we all need basic knowledge in cardiovascular morphology for being able to understand what we see, hear, or read. That is why the recently published AEPC training guidelines (2) emphasize the relevance of training in cardiovascular morphology to all who care for patients with cardiovascular malformations.

## Author Contributions

The author confirms being the sole contributor of this work and has approved it for publication.

## Conflict of Interest

The author declares that the research was conducted in the absence of any commercial or financial relationships that could be construed as a potential conflict of interest.

## Publisher's Note

All claims expressed in this article are solely those of the authors and do not necessarily represent those of their affiliated organizations, or those of the publisher, the editors and the reviewers. Any product that may be evaluated in this article, or claim that may be made by its manufacturer, is not guaranteed or endorsed by the publisher.
